# A SNARE-like protein from *Solanum lycopersicum* increases salt tolerance by modulating vesicular trafficking in tomato

**DOI:** 10.3389/fpls.2023.1212806

**Published:** 2023-08-01

**Authors:** Josselyn Salinas-Cornejo, José Madrid-Espinoza, Isabel Verdugo, Lorena Norambuena, Simón Ruiz-Lara

**Affiliations:** ^1^ Laboratorio de Genómica Funcional, Instituto de Ciencias Biológicas, Universidad de Talca, Talca, Chile; ^2^ Departamento de Biología, Facultad de Ciencias, Universidad de Chile, Santiago, Chile

**Keywords:** SNARE-like superfamily protein, vesicular trafficking, *Solanum lycopersicum*, endocytosis, sodium compartmentalization, salt stress

## Abstract

Intracellular vesicular trafficking ensures the exchange of lipids and proteins between endomembrane compartments. This is relevant under high salinity conditions, since both the removal of transporters and ion channels from the plasma membrane and the compartmentalization of toxic ions require the formation of vesicles, which can be maintained as multivesicular bodies or be fused to the central vacuole. SNARE proteins (Soluble N-ethylmaleimide-sensitive factor attachment receptor) participate in the vesicle fusion process and give specificity to their destination. Plant genome studies have revealed a superfamily of genes that encode for proteins called SNARE-like. These proteins appear to be participating in vesicular trafficking with similar functions to those of SNARE proteins. A SNARE-like, named SlSLSP6, in *Solanum lycopersicum* plants has been shown to be induced under high salinity conditions. A phylogenetic relationship of SlSLSP6 with SNARE-like proteins of salinity-tolerant plants, including *Salicornia brachiata*, *Zostera marina* and *Solanum pennelli*, was determined. Considering its amino acid sequence, a putative clathrin adapter complex domain and palmitoylation site was predicted. Subcellular localization analysis evidenced that SlSLSP6 is mostly localized in the plasma membrane. Using transgenic tomato plants, we identified that overexpression of *SlSLSP6* increased tolerance to salt stress. This tolerance was evident when we quantified an improvement in physiological and biochemical parameters, such as higher chlorophyll content, performance index, efficiency of photosystem II and relative water content, and lower malondialdehyde content, compared to control plants. At the subcellular level, the overexpression of *SlSLSP6* reduced the presence of H_2_O_2_ in roots and increased the compartmentalization of sodium in vacuoles during salt stress. These effects appear to be associated with the higher endocytic rate of FM4-64, determined in the plant root cells. Taken together, these results indicate that SlSLSP6 increases tolerance to salt stress by modulating vesicular trafficking through over-induction of the endocytic pathway. This work contributes to understanding the role of this type of SNARE-like protein during salt stress and could be a potential candidate in breeding programs for tolerance to salt stress in tomato plants.

## 1 Introduction

In eukaryotes, the endomembrane system is composed of a series of compartments that are interconnected through diverse and specific trafficking pathways (Saito and Ueda, 2009; Pizarro and Norambuena, 2014). The direction of transported cargo and its location depend on the balance of all the pathways that affect its movement. The balance of the different pathways will determine the amount of protein in each of the compartments. The ability to change this balance could be useful when the presence of certain proteins changes in a particular compartment in response to a stimulus or disturbance (Uemura and Ueda, 2014; Zhao et al., 2020), as observed in plants subjected to salt stress conditions (Baral et al., 2015a; Baral et al., 2015b). Since the transport of lipids and proteins between the different cellular compartments is carried out mainly through membranous vesicles, the regulation of intracellular vesicular traffic becomes highly important during salt stress, since the cellular requirements must be maintained to cope with the stress that threatens the viability of the plant (Pizarro and Norambuena, 2014). In plants exposed to high salinity, an osmotic and ionic imbalance occurs inside their cells that compromises physiological, biochemical, and molecular activities and leads to a loss of plant viability (Macková et al., 2013). Removal of transporters and ion channels from the plasma membrane through the process of endocytosis, followed by degradation in vacuoles, has been suggested as a common mechanism to prevent toxicity in plants under salt stress conditions (Baral et al., 2015a; Ivanov and Vert, 2021). Most of the endocytosed cargo converges in the vacuole and is subjected to degradation, another part is recycled back to the plasma membrane and a less common part is reoriented and maintained in prevacuolar structures. For this to happen, a series of proteins are involved in the anchoring and fusion of vesicles, for the recognition and direction of membrane fusion (Barlow and Dacks, 2018; Shimizu and Uemura, 2022). Among them, it has been described that SNARE and SNARE-like proteins play a critical role in this process (Saito and Ueda, 2009; Singh et al., 2016; Rosquete and Drakakaki, 2018). Therefore, its function is essential in these multiple vesicular trafficking routes involved during saline stress (Saito and Ueda, 2009; Pizarro and Norambuena, 2014).

It has been reported that overexpression of *GsSNAP33*, a gene that codes for a protein of the plasma membrane Qb+Qc-SNARE type, in *Arabidopsis thaliana*, significantly increases tolerance to salt stress (Nisa et al., 2017). Additionally, it has been identified that the overexpression of the *AtSFT12* gene, which codes for a Qc-SNARE located in the Golgi complex, confers tolerance to salt stress in *A. thaliana* plants, since the overexpressing plants accumulate a greater amount of sodium in the vacuole (Tarte et al., 2015). Also, the silencing of two genes for vacuole-localized SNAREs, *VAMP711* and *SYP22*, increase tolerance to salt stress through compartmentalization of reactive oxygen species (ROS) and sodium ions in multivesicular bodies, respectively (Leshem et al., 2006; Hamaji et al., 2009). Indeed, in the mutants of *vamp711* and *syp22*, the fusion of vesicles with the vacuole would be blocked. In *vamp711*, the tolerance to salt stress has been associated with the endocytosis of plasma membrane NADPH oxidases (e.g., RbohD, among others), resulting in the production of hydrogen peroxide inside the vesicles, preventing the mobilization of ROS towards the vacuole (Leshem et al., 2006). Although, ROS play a fundamental role under many stresses since they activate different regulatory pathways to promote tolerance (Hao et al., 2014). Under salt stress, excessive levels of these induces programmed cell death (Apel and Hirt, 2004). To counteract this and generate tolerance to salt stress, RbohD is removed from the plasma membrane and compartmentalized in multivesicular bodies to maintain lower levels of cytoplasmic ROS (Martinière et al., 2019). This reduction of ROS decreases the oxidative damage of the vacuole and prevents its alkalinization. Therefore, this would promote the functioning of the H^+^ gradient and the vacuolar sequestration of excess cytoplasmic sodium (Leshem and Levine, 2007; Baral et al., 2015b).

Despite its agronomic importance, studies on vesicular traffic in *Solanum lycopersicum* plants are not very extensive, which makes it a potential plant model to study salt stress response genes. Recently, the identification and transcriptional analysis of tomato SNARE proteins in response to salt stress were determined. Among them, *SlVAMP721.2*, *SlSYP71.1* and *SlSNAP33.2* showed strong induction (Salinas-Cornejo et al., 2019). Hence, they could be considered as future candidates in studies associated with abiotic stress. Precisely, the evaluation of *SlSNAP33.2* showed to improve the tolerance to salt stress by increasing the rate of endocytosis and the physiological and biochemical parameters of tomato plants grown under greenhouse conditions (Salinas-Cornejo et al., 2021). Regarding, the SNARE-like proteins in tomato are unknown. Recent studies showed that a *SNARE-like* gene, *SbSLSP*, that codes a plasma membrane protein of the halophytic plant *Salicornia brachiata*, confers tolerance to salt stress when is overexpressed in tobacco plants. *SbSLSP* in transgenic plants maintains cellular homeostasis, increases the detoxification of ROS, and improves physiological and biochemical parameters, under stress conditions (Singh et al., 2016). Even though a model of intracellular functioning of this protein was proposed, the characterization of this functionality has not been demonstrated. The question is whether this tolerance is generated by being SNARE-like from a halophyte plant or by the intracellular behavior of this type of protein. Advance knowledge of the intracellular role played by SNARE-like proteins under salt stress conditions would be essential to answer this question. According to this, we studied the effects of overexpression of a *SNARE-like* gene on vesicular trafficking in *S. lycopersicum* plants subjected to high salinity conditions. Consequently, the putative gene that codes for a SNARE-like in the genome of *S. lycopersicum* was identified and its expression levels during salt stress were analyzed. Then, the effects of its overexpression on salt stress tolerance in *S. lycopersicum* plants were evaluated by measuring physiological and biochemical parameters. Subsequently, the subcellular localization of SNARE-like was determined, to then analyze the endocytic rate, the accumulation of hydrogen peroxide and the vacuolar sodium content under salt stress conditions. These results allow us to suggest that the tolerance conferred by these SNARE-like proteins is due to the modulation of intracellular vesicular traffic induced by a high endocytic rate. In addition, it will serve as a basis to carry out evaluations on the use of this type of genes in programs associated with genetic improvement and tolerance to salt stress in tomato plants.

## 2 Materials and methods

### 2.1 Plant material and culture conditions

For this research, we used tomato seeds (*Solanum lycopersicum* cv. Moneymaker) which were sown in a 0.5 L plastic pot with a mixture of 1:1:1 of perlite, vermiculite, and peat as substrate. After 15 days post germination, seedlings were transferred to 2 L plastic pots containing the same substrate. The plants were watered once a week with a nutrient solution containing 1.1 g/L of Murashige and Skoog salts (MS; Murashige and Skoog, 1962). Tomato plants were cultivated and propagated in growth chambers at 25°C with a long-day photoperiod (16/8 hours light/dark). After 10-12 weeks, tomato plants were subjected to saline stress treatments by adding 400 mL of 300 mM NaCl for 72 hours. Three plants for each condition were considered. Leaf and root were collected separately for each plant in the periods 0, 3, 6, 12, 24, 48 and 72 hours of treatment with salt stress, to measure transcript levels. The collected tissue was quickly frozen with liquid nitrogen and stored at -80°C for further analysis.

### 2.2 *In silico* analysis of the genes that code for SNARE-like in tomato plants

The Solgenomic database (http://www.solgenomics.net) was used to search for *S. lycopersicum* protein displaying sequence identity with the SNARE-like protein from *S. brachiata* (accession number KF111691). The sequence of the gene *Solyc06g068080.2* (*SlSLSP6*) was selected by the highest score of similarity of the gene product. Sequences were aligned using the MUSCLE software (https://www.ebi.ac.uk/Tools/msa/muscle/). Comparative analyses were performed for each protein with the BLASTp program (http://www.ncbi.nlm.nih.gov), InterPro (https://www.ebi.ac.uk/interpro/) and Pfam databases. A phylogenetic tree was built using the MEGA7 (http://www.megasoftware.net) (Kumar et al., 2016) with the neighbor-joining method (Saitou and Nei, 1987) and bootstrap analysis of 1000 replications.

### 2.3 Isolation and purification of total RNA and cDNA synthesis

Total RNA was extracted from leaves and roots of *S. lycopersicum* plants using the SV Total RNA Isolation System commercial kit (Promega, Madison, WI, USA). Each sample was treated with DNase I (Ambion^®^ TURBO DNA-free™) to remove remaining genomic DNA. RNA concentration and purity were estimated in a NanoQuant spectrophotometer (UV-160A, Kyoto, Japan).

First-strand cDNA synthesis was performed with 1–2 μg of isolated RNA using the Maxima H Minus First Strand cDNA Synthesis kit (Thermofisher, Massachusetts, USA). cDNA samples were stored at -20°C.

### 2.4 PCR and quantitative real-time PCR (RT-qPCR)

The PCR reactions were carried out in a final volume of 25 μL which contained DNA, 1X PCR buffer, MgCl_2_, dNTPs, the corresponding oligonucleotides (**Supplementary Table 1**) and the Taq polymerase enzyme (Promega Corporation). The PCR product was analyzed in agarose gel electrophoresis (1X TAE Buffer). RT-qPCR analysis was carried out using the Brilliant SYBR Green qPCR MasterMix System (Stratagene, La Jolla, CA) according to the manufacturer’s instructions on a DNA engine Opticon 2 Cycler System (MJ Research, Watertown, MA). For each sample, RT-qPCR was carried out in triplicate (technical replicates). The 2^−ΔΔCt^ method was applied to calculate the fold change of gene transcript levels (Livak and Schmittgen, 2001). *Ubiquitin3* gene (accession X58253) was used as a housekeeping gene (Yáñez et al., 2009).

### 2.5 Gene transformation of tomato plants

For the transformation of tomato plants, the coding sequence of *SlSLSP6* was amplified from cDNA of leaves using the primers described in **Supplementary Table 1**. After the amplification reaction, the DNA fragments were cloned into pGEM-T vector (Promega) and sequenced. Then, the complete cDNA of the *SlSLSP6* was inserted between the XbaI-BamHI restriction sites, replacing *β-glucuronidase* gene (GUS gene) of the binary vector pBI121 (Clontech, Palo Alto, CA, USA), and staying under the control of the constitutive promoter CaMV 35S, generating the 35S::SlSLSP6 cassette. Tomato plants were transformed with the 35S::SlSLSP6 cassette and with the unmodified pBI121 for control plants (Vector). Chemo-competent *Agrobacterium tumefaciens* GV3101 was cultured in YM solid medium (0.04% w/v yeast extract, 1% w/v mannitol, 1.7 mM NaCl, 0.8 mM MgSO_4_.7H_2_O and 1.5 w/v of agar) and grown at 28°C for 48 hours with the followed antibiotics: kanamycin (50 mg/mL), gentamicin (100 mg/mL) and rifampin (100 mg/mL). The colonies were analyzed by PCR. A culture of the PCR-confirmed strains was used to transform tomato cotyledons from 10-day-old seedlings, according to the method of Fillati et al. (1987). After six months of organogenesis, the shoots obtained were individualized. The plants were selected in kanamycin antibiotic (50 mg/mL) and characterized by PCR and RT-qPCR. Sixteen *SlSLSP6* overexpressor lines were selected (L1-L16).

### 2.6 Determination of performance and tolerance to salt stress

*SlSLSP6* overexpressing plants (L9 and L10) and control plants (WT and Vector) with similar size and number of leaves were subjected to salt stress conditions grown in pots with perlite and vermiculite substrate (1:1). These plants were subjected to irrigation with 200 mL of a 300 mM NaCl saline solution every 72 hours. Plant performance and tolerance parameters were evaluated after 5, 15 and 25 days of stress treatment. Each time of the experimental trial considers 3 plants as biological replicates. Relative water content (RWC) was estimated according to the modified protocol of Maggio et al. (2007) and Vasquez-Robinet et al. (2008), where RWC = [(FW – DW)/(TW – DW)] x 100. Three leaves of about the same size and location on the plant were weighed (fresh weight, FW) and then incubated in distilled water for 24 hours at room temperature in darkness. Then, the tissue was weighed to calculate the turgid weight (TW) and finally, the samples were dried at 60°C for 48 hours for the determination of the dry weight (DW). Additionally, the efficiency of photosystem II represented as Fv/Fm and the performance index (PI) were evaluated. These measurements were obtained using a pocket fluorometer (Hansatech, King’s Lynn, Norfolk, England) following the procedure described by Giorio (2011). The chlorophyll content was evaluated in leaf discs following the previously described protocols (Orellana et al., 2010; Salinas-Cornejo et al., 2021). According to this, the leaf discs were ground with 80% acetone (v/v) and the extract was centrifuged. The absorbance of the supernatant was measured using the NanoQuant spectrophotometer (UV-160A, Kyoto, Japan), using the method described by Lichtenthaler and Wellburn (1983).

To determine lipid damage, the lipid peroxidation marker (MDA) was determined according to the modified protocol of Dionisio-Sese and Tobita (1998) and Ghanem et al. (2008). The concentration was obtained by subtracting the absorbance between 600 and 532 nm. Then, the value was multiplied using the molar extinction coefficient of 155 mmol^-1^cm^-1^. In addition, the ROS accumulation was observed in cells of the apical zone of the root (root length of 0.5 cm) of transgenic tomato plants (L9 and L10 lines) and control plants (WT and Vector). Tomato seedlings exposed for 40 minutes to liquid MS medium supplemented with 200 mM NaCl were subsequently incubated for 20 minutes with the fluorescent tracer H_2_DCFDA 10 μM (2’,7’-dichlorodihydrofluorescein diacetate, ThermoFisher Scientific, Massachusetts, USA) as described by Leshem et al. (2006) and Zhao et al. (2009). To delimit the plasma membrane of each cell, the seedlings were stained with FM4-64 5 μM for 15 minutes in darkness at 4°C. Then, the roots were visualized immediately.

### 2.7 SlSLSP6 subcellular localization

The subcellular accumulation of SlSLSP6 was performed in 10-day-old *Arabidopsis thaliana* seedling lines expressing different organelle markers described by Geldner et al., 2009. Each line was transiently transformed using the 35S::GFP-SlSLSP6 construct, contained in the pAM1 vector. The *SlSLSP6* gene, amplified as described in section 2.5, was inserted into the pAM1 vector in the C-terminal orientation of the GFP gene. We use an adaptation of the technique carried out by Wu et al. (2014), called AGROBEST. The Arabidopsis marker lines RabG3f-mCherry (Germplasm ID CS781670; late endosome/tonoplast), RabF2a-mCherry (Germplasm ID CS781672; late endosome/prevacuolar compartments), VAMP711-mCherry (Germplasm ID CS781673; tonoplast), VTI12-mCherry (Germplasm ID CS781675;early endosome/TGN), SYP32-mCherry (Germplasm ID CS781677; Golgi complex) and PIP1;4-mCherry (Germplasm ID CS781687; plasma membrane) were grown in similar MS agar plates in growth chambers.

### 2.8 Endocytosis rate analysis

The rate of plasma membrane internalization was determined in root apical zone cells (root length 0.5 cm) of both transgenic tomato seedlings (L9 and L10 lines) and control plants (WT and Vector) using the membrane tracer FM4-64 ( Molecular Probes, Eugene, OR). Roots were stained with FM4-64 (5 μM) in a solution of MS liquid medium at 4°C for 20 minutes in darkness. At 5-, 15-, and 30-minutes at room temperature, different seedlings were by imaged. To determine the effect of salt stress on the endocytic rate, a group of seedlings, previously stained with FM4-64 (5μM), was subjected to salt stress by incubation with liquid MS medium supplemented with 200 mM NaCl for 30 minutes at room temperature.

### 2.9 Analysis of intracellular sodium accumulation

The accumulation of sodium into the vacuole was determined using the Na^+^ indicator Sodium Green™ (ThermoFisher Scientific, Massachusetts, USA). Tomato seedlings with root length of 0.5 cm were incubated in MS medium supplemented with 200 mM NaCl for a period of 16 hours. After treatment, the seedlings were incubated with 5 μM Sodium Green™ for 2 hours and a non-ionic surfactant Pluronic F-127 20% w/v (ThermoFisher Scientific, Massachusetts, USA) to improve the uptake of the dye, following an adaptation of the method described by Tarte et al., 2015 (San Martín-Davison et al., 2017; Salinas-Cornejo et al., 2021). Then, the roots were stained for 20 minutes with FM4-64 5 μM (in the dark at 4°C) to delimit the plasma membrane and visualized.

### 2.10 Quantification of fluorescent markers

To quantify colocalization data, colocalization between GFP-SlSLSP6 and mCherry markers was quantified using the square of Pearson correlation coefficient (R^2^). The colocalization between GFP-SlSLSP6 and PIP1;4-mCherry was scored from a defined region of interest (ROI) for calculation of the Pearson correlation coefficient. R^2^ was estimated considering 10 images using the Zeiss Zen 2010 software (zeiss.com).

The FM4-64 fluorescence was quantified manually at the intracellular and plasma membrane compartments using ImageJ software (http://rsb.info.nih.gov/ij/). FM4-64 internalization rate was determined as the ratio of both signals in each cell, using the equation described by Varma and Mayor (1998) and Baral et al. (2015a).

The quantification of the signal of H_2_DCFDA and Sodium Green markers was performed using ImageJ software. The mean of fluorescence was scored from a manually defined ROI in each image. Images were processed using a color-coded intensity, with the lowest fluorescence intensity corresponding to blue and highest fluorescence intensity corresponding to green color.

To quantify the fluorescence intensities, laser, gain and pinhole settings of the confocal microscope were identical and constant among different treatments. All confocal experiments were independently repeated at least three times, considering for each experiment at least 25 cells from 5 roots of 5 different plants for each genotype.

### 2.11 Confocal microscopy

Root cells were visualized with Zeiss-LSM 710 confocal microscope (Zeiss Axio Observer Z1 equipped with LSM700 module and PTM detector). An Fluar 40x/1.30 oil M27 objective or N-Achroplan 10x/0.25 M27 lens were used. Digital zoom 1X or 4X. The parameters were: laser 555 nm/emission>588–596 nm for FM4-64 or fluorescent protein mCherry, laser 488 nm/emission 517–561 nm for the fluorescent protein GFP, Sodium-Green or H_2_DCFDA (filter SP 555 Zeiss), pixel dwell of 25.2 μs, 488/555 nm lasers set at 2%, pinhole diameter of 70 μm, and beam splitter MBS 405/488/555/639.

### 2.12 Statistical analysis

Statistical analyzes were performed using the R program (version 1.7). Statistical significance was determined by one-way ANOVA analysis (p < 0.05).

## 3 Results

### 3.1 Identification of a SNARE-like superfamily gene from *Solanum lycopersicum*

Using the Solgenomics database, the sequence coding for a putative SNARE-like in *Solanum lycopersicum* was identified. This gene was named *SlSLSP6* (Solyc06g068080.2), which has not been previously characterized. The analysis using NCBI Blastp tool indicated that the amino acid sequence of SbSLSP from *Salicornia brachiata* (Accession AGV05388) displayed an 84.35% identity to the sequence SlSLSP6 from *Solanum lycopersicum* (Accession XP_010322672). SlSLSP6 is a protein of 147 amino acids with no transmembrane domains in its structure as expected for a SNARE protein. However, SlSLSP6 displayed putative palmitoylation site at the cysteine residue located at position 115 (Cys115) at the LDKYGKICLCLDEIV motif that may allow it to interact with membranes (**Supplementary Figure 1**). Multiple alignment and comparison of the amino acid sequences of SlSLSP6 with SNARE-like from *Arabidopsis thaliana* (AtSLSP), *Salicornia brachiata* (SbSLSP) and two other sequences identified in *Solanum lycopersicum* (SlSLSP3 and SlSLSP11) revealed a high identity score as well as the presence of conserved domains as a SNARE-like superfamily protein (**Supplementary Figure 1**). The phylogenetic analysis of plants SNARE-like proteins showed that SlSLSP6 clustered with proteins from the halophytic plants *S. brachiata* and *Zostera marina* (Park et al., 2016; Singh et al., 2016) (**Supplementary Figure 2**). SISLSP6 is grouped also with the proteins from *Nicotiana tomentosiformis* and *Solanum pennelli*, considered salt-tolerant plants. Consequently, based on the high sequence identity and the presence of the structural domains of the SNARE-like superfamily proteins, the SlSLSP6 is predicted to function as a SNARE-like protein in *S. lycopersicum*.

### 3.2 *SlSLSP6* responds to salt stress

**Figure 1:**
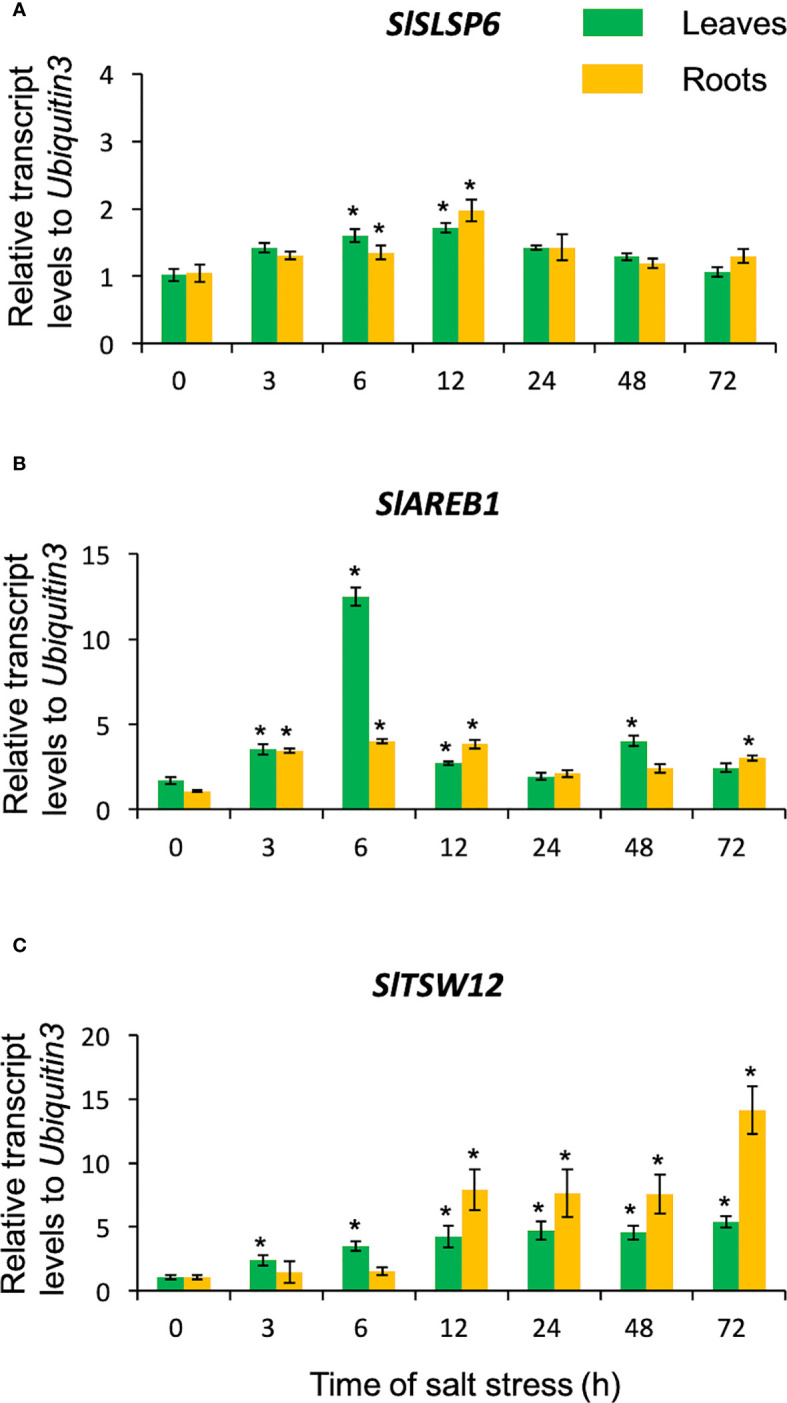
Transcript levels of *SlSLSP6* in *S. lycopersicum* exposed to salt stress. Ten-twelve-week-old tomato plants were watered with 300 mM NaCl solution for 3, 6, 12, 24, 48 and 72 hours. Roots and leaves were collected separately for RNA isolation. The transcript levels of *SlSLSP6* **(A)**, *SlAREB1* **(B)** and *SlTSW12* **(C)** were measured by RTqPCR. Results are expressed as relative expression considering *Ubiquitin3* as the gene of reference. Mean and standard errors of three biological replicates are shown. Asterisks above the bars indicate statistically significant difference to the level before starting the salt treatment (time 0) determined by one-way ANOVA (p-value < 0.05).

The *S. lycopersicum* plants were subjected to saline stress (300 mM NaCl) for 72 hours, to evaluate whether the *SlSLSP6* responds to such a challenge (**Figure 1**). The salt treatment indeed was a stressful condition since the salt stress-marker-genes *AREB1* (Orellana et al., 2010; Madrid-Espinoza et al., 2019) and *TSW12* (Torres-Schumann et al., 1992; San Martín-Davison et al., 2017) responded as expected, increasing their transcript level along the treatment (**Figures 1B, C**). Interestingly, *SlSLSP6* transcript levels increased at 3 hours of salt stress in both roots and leaves. This increase was maintained as far as 12 hours of treatment (**Figure 1A**). After 24 hours of salt stress, the *SlSLSP6* transcript level becomes similar to the beginning of the trial (time 0) in both tissues. These results indicate that *SlSLSP6* is induced under salt stress in leaves and roots suggesting the participation of the *SlSLSP6* gene product in the primary response of *S. lycopersicum* to saline stress.

### 3.3 The overexpression of *SlSLSP6* in *S. lycopersicum* confers tolerance to salt stress

To evaluate the participation of *SlSLSP6* in the response to salt stress, we study the effects of its overexpression in *S*. *lycopersicum*. Then, cDNA of the *SlSLSP6* gene was cloned into the binary vector pBI121 to express the coding sequence under the 35S promoter in plants. *S. lycopersicum* cv. Moneymaker was transformed mediated by *Agrobacterium tumefaciens* and transformant explants were selected by kanamycin resistance and verified by PCR. The same protocol was followed transforming tomato plants with the empty vector to be used as a control (Vector) for the following experiments. The expression levels of *SlSLSP6* were evaluated by qRT-PCR on the *SlSLSP6* transformant plants. Three lines were identified as overexpressors of *SlSLSP6* (**Supplementary Figure 3**). Two of them, L9 and L10 were selected for the further characterization.

**Figure 2:**
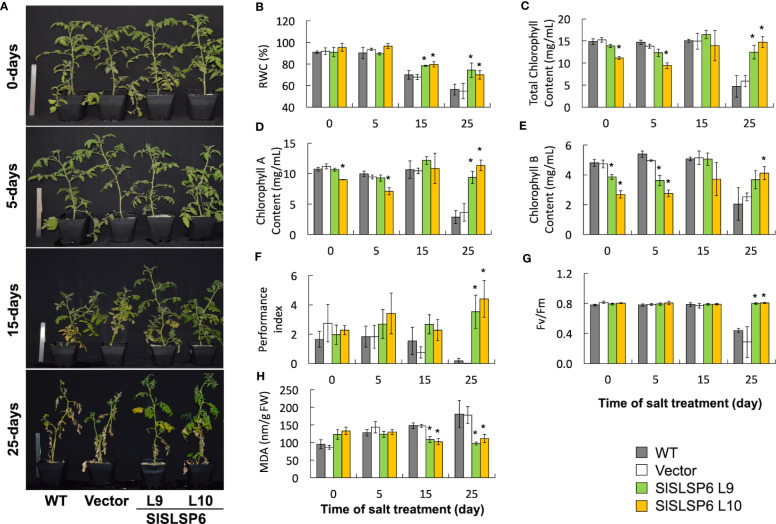
The response of plants transformed with *SlSLSP6* shows a greater tolerance to high salinity conditions. Tomato plants (WT, Vector and *SlSLSP6* overexpression lines) were watered with 300 mM NaCl solution every 72 hours for a period of 25 days. Plants performance and tolerance parameters were evaluated at the beginning of the trial (time 0) and after 5, 15 and 25-days of salt stress. **(A)** Representative images of each line of tomato plants. **(B)** Relative water content (RWC). **(C)** Total chlorophyll content. **(D)** Chlorophyll A content. **(E)** Chlorophyll B content. **(F)** Performance Index (PI). **(G)** PSII Maximum quantum efficiency analysis (Fv/Fm). **(H)** Malondialdehyde (MDA) content. Mean and standard error of three plants in three independent experiments are shown. Asterisks indicate statistically significant differences between WT and transgenic plants (p-value < 0.05) determined by one-way ANOVA.

**Figure 3:**
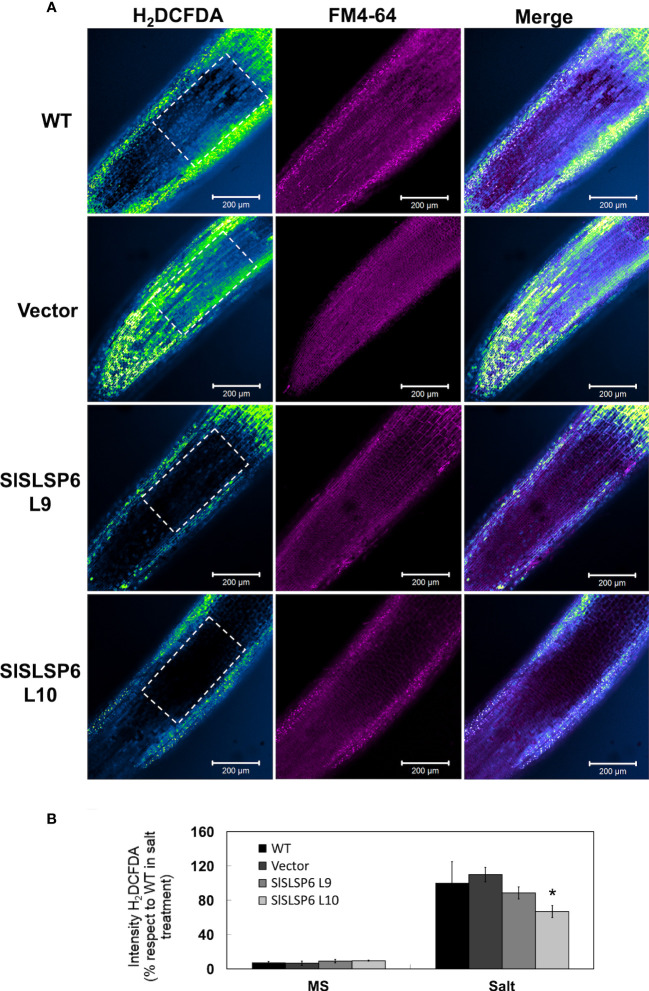
H_2_O_2_ content in *Solanum lycopersicum* roots under salt stress conditions. **(A)** Representative images of H_2_O_2_ production in control tomato seedlings and *SlSLSP6* overexpressing lines under 200 mM NaCl and observed by confocal microscopy. The ROI delimited by the white dashed lines was considered for quantification. Tomato roots were treated with H_2_DCFDA (color-coded intensity, with the lowest fluorescence intensity corresponding to blue and highest fluorescence intensity corresponding to green color) and FM4-64 tracer (in magenta). Images of the differentiation and elongation zones of the roots were obtained. The bar indicates a scale of 200 µm. **(B)** Quantification of fluorescence intensity of H_2_DCFDA. The values ​​indicate the percentage relative to 100% fluorescence intensity evaluated in the WT plants under salt stress. Data indicate mean values ​​and standard errors. Asterisks indicate statistically significant differences between control lines and transgenic plants (p-value < 0.05), using one-way ANOVA.

The response to salt stress of *SlSLSP6* overexpressor plants was evaluated and compared to control plants as well as wild type plants. Two-month-old tomato plants were irrigated every 72 hours with a solution of 300 mM NaCl. Plant performance parameters were evaluated just before starting the trial and after 5, 15 and 25 days of salt treatment. After 15 days of treatment, the macroscopic symptoms as yellow leaves induced by salt stress began to appear in wild type and Vector plants; however, they are less abundant on L9 and L10 lines. After 25 days of salt stress, there were evident differences between the control plants and L9 and L10 plants. The symptoms of chlorosis and wilting were dramatic on WT and Vector plants showing a significant loss of the older leaves. However, L9 and L10 plants exhibited less chlorotic leaves and displayed several green leaves after the same period of salt stress treatment, suggesting an increase of salt tolerance due to the higher level of *SlSLSP6* gene product (**Figure 2A**). Consistently, the RWC of L9 and L10 lines reached 74% and 64%, respectively while in wild type plants decreased to 56% after 25 days of salt stress (**Figure 2B**). This suggests that there is greater turgidity in the leaves of the overexpressing lines, which is consistent with the better appearance of the plants (**Figure 2A**). The applied salt stress treatment resulted in 70% reduction of chlorophyll levels in wild type and Vector plants. However, the overexpression of *SlSLSP6* rescued the drop on chlorophyll (chlorophyll A and chlorophyll B) content induced by 25 days of salt stress (**Figures 2C–E**). These results suggest that the high levels of *SlSLSP6* somehow increase the plant tolerance. To confirm that *SlSLSP6* overexpression plants are more tolerant to salt stress, we evaluate physiological state of the plants by measuring the performance index (PI) and the efficiency of photosystem II (PSII), represented by the maximum quantum efficiency of the PSII (Fv/Fm). The PI values in the *SlSLSP6* overexpressor lines were significantly higher compared to control plants, which decreased by approximately 75% after 25 days of stressful conditions (**Figure 2F**). As expected, the ratio Fv/Fm dropped drastically to 0.4 in control plants after 25 days of salt stress (**Figure 2G**). In contrast, *SlSLSP6* overexpressors lines kept the levels of Fv/Fm as plants without any salt stress condition. Consistently, there was no membrane damage on overexpressor plants as result of the salt stress while in control plants lipid peroxidation increases along the salt treatment (1.8 times higher after 25 days) (**Figure 2G**). In addition, we used the fluorescent marker H_2_DCFDA to determine the level of ROS in tomato roots under normal conditions (**Supplementary Figure 4**) and subjected to 200 mM NaCl. The L9 line showed to be less tolerant since it had a similar result to the control plants. Instead, the L10 line showed 15 - 30% decrease of ROS accumulation compared to control plants (**Figures 3A, B**). Overall, the overexpression of *SlSLSP6* prevents the detrimental effects of salt stress improving plant performance at the physiological and cellular level. These results demonstrate that the overexpression of *SlSLSP6* improves the tolerance of the tomato plants to high salinity conditions.

### 3.4 SlSLSP6 is localized at the plasma membrane of root cells of *A. thaliana*

**Figure 4:**
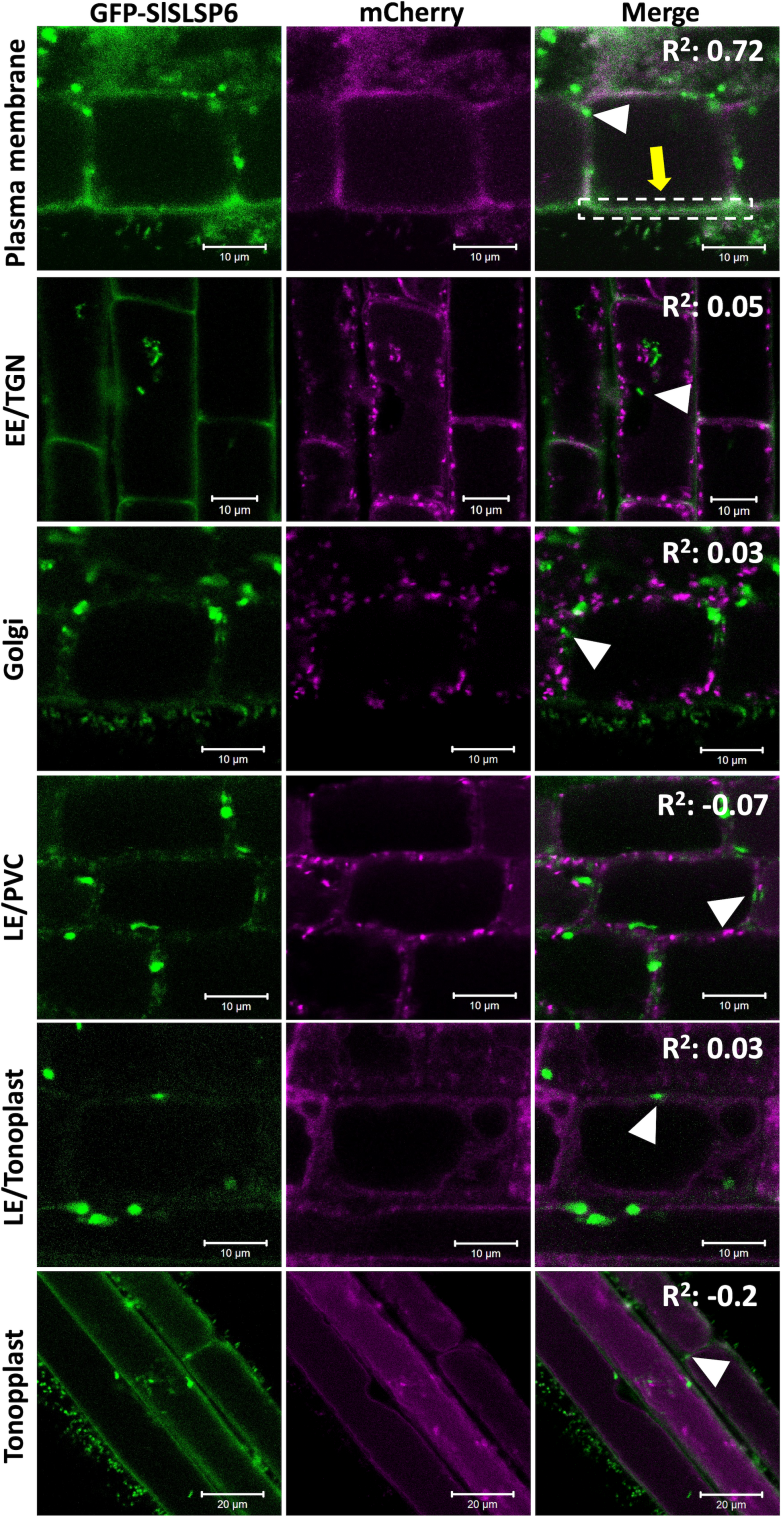
SlSLSP6 resides in the plasma membrane of *A. thaliana* root cells. 10-day-old *Arabidopsis thaliana* mCherry-marker lines of different endomembrane compartments were transiently transformed with the vector that allows to express a GFP-fusion to SlSLSP6 version. GFP and mCherry (in magenta) fluorescence in root cells were visualized by confocal microscopy. For each marker line, 25 cells of 15 plants were evaluated. Images of the differentiation or elongation zone of the roots were obtained. The square of Pearson correlation coefficient is indicated (R^2^). In the colocalization with plasma membrane, the ROI delimited by the white dashed lines was considered for quantification. Markers: Plasma membrane, PIP1;4-mCherry; EE/TGN, VTI12-mCherry; Golgi, SYP32-mCherry; LE/PVC, RabF2a-mCherry; LE/tonoplast, RabG3f-mCherry; Tonoplast, VAMP711-mCherry.

SbSLSP from *S. brachiata*, a SNARE-like protein, is localized within the endomembrane system (Singh et al., 2016). Similar to SbSLSP, SlSLSP6 from *S. lycopersicum* would have a conserved function in the endomembrane system. Although SlSLSP6 does not display a transmembrane domain most likely it is able to be recruited to membranes in order to accomplish its function. To determine the subcellular localization of SlSLSP6, we overexpress transiently a GFP-SlSLSP6 construct in a collection of *A. thaliana* that express fluorescent endomembrane compartment markers (Geldner et al., 2009). The following mCherry markers were selected: plasma membrane (AtPIP1;4), early endosome/TGN (AtVTI12), Golgi complex (AtSYP32), late endosome/prevacuolar compartments (AtRabF2a), late endosome/tonoplast (AtRabG3f) and tonoplast (AtVAMP711). *A. thaliana* roots were transiently transformed using a protocol adapted from the AGROBEST technique (Wu et al., 2014; Wang et al., 2018), with two different constructions: the plasmid pAM1 carrying the sequence to express GFP : SlSLSP6 (35S::GFP-SlSLSP6) or with the empty vector (35S::GFP), as a negative control. The results indicated that GFP-SlSLSP6 signal overlapped with the mCherry AtPIP1;4 protein, located in the plasma membrane (yellow arrow **Figure 4** and **Supplementary Figure 5**), strongly suggesting that SlSLSP6 is accumulated in the plasma membrane. In contrast, the GFP-SlSLSP6 distribution did not colocalize with the markers of the vacuole and Golgi complex. In addition to the plasma membrane distribution, GFP-SlSLSP6 displayed a punctate pattern (white arrowheads **Figure 4**) with similar characteristics to endosomes or PVCs. However, these bodies did not colocalize with the AtVTI12 nor AtRabF2a proteins, indicating there are different compartments than the early endosome/TGN nor LE/PVC. Therefore, these results suggest that the SlSLSP6 protein would be participating in processes linked to the plasma membrane, such as endocytosis, and may participate in pathways to the early or late endosome.

### 3.5 The overexpression of *S. lycopersicum SlSLSP6* increases the endocytosis under normal and salt stress conditions

**Figure 5:**
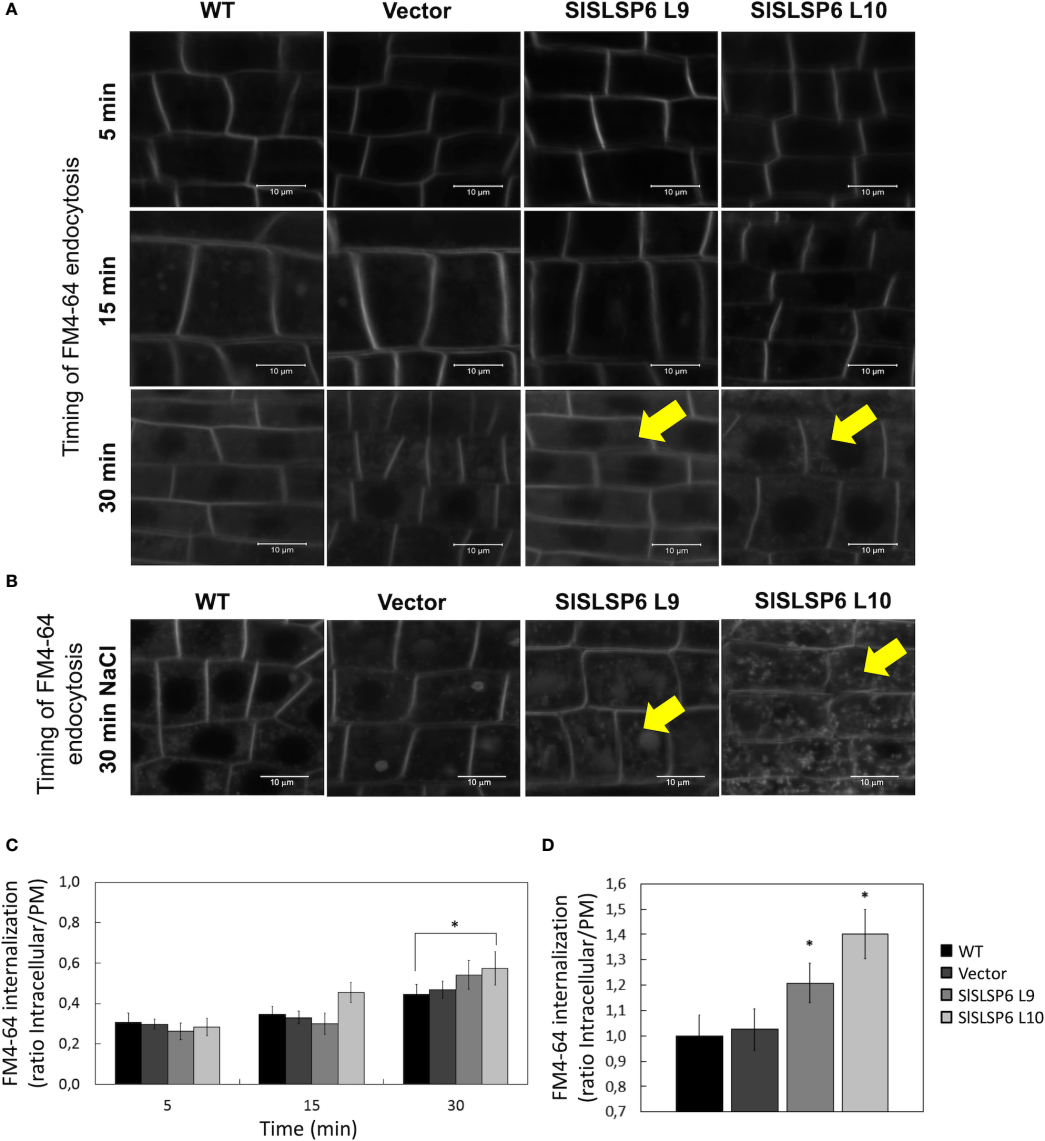
Endocytosis in wild type and transgenic *Solanum lycopersicum* roots. The endocytosis rate was evaluated on root cells with root length of 0.5 cm from WT, Vector and tomato plants that overexpress *SlSLSP6* (L9 and L10 lines) using the FM4-64 tracer. **(A)** Internalization of FM4-64 (in grayscale) was evaluated by confocal microscopy at 5, 15 and 30 minutes. Representative images of the differentiation zone of the root of three independent experiments (n=25 cells) are shown. Yellow arrows indicate representative FM4-64-stained endosomes. **(B)** FM4-64 internalization (in grayscale) on root plants treated previously with 200mM NaCl. Yellow arrows indicate representative FM4-64-stained endosomes. **(C)** Internalization rate of FM4-64 was evaluated in the experiment on **(A)** Mean and standard error are shown; n=25 cells. Asterisks indicate statistically significant differences between WT and transgenic plants (p-value < 0.05), using one-way ANOVA. **(D)** FM4-64 internalization rate of plants in **(B)** Results are expressed as the ratio between intracellular and plasma membrane signal. Mean and standard errors are shown. Asterisks indicate statistically significant differences between WT and transgenic plants (p-value < 0.05), using one-way ANOVA.

Endocytic trafficking has been proved to be important for salt stress tolerance (San Martín-Davison et al., 2017). In fact, an increased rate of endocytosis has been shown to increase plant tolerance under salt stress conditions (Salinas-Cornejo et al., 2021). As SlSLSP6 could be participating in processes linked to the plasma membrane, we hypothesized that this SNARE-like would have an active role in endocytosis. To test the rate of endocytosis, the internalization of the tracer FM4-64 was analyzed in *SlSLSP6* overexpression lines (L9 and L10) and compared to wild type and Vector tomato seedling roots. The L10 overexpressing line grown in control conditions displayed a higher internalization rate than control plant: FM4-64 internalization after 15 and 30 minutes increased 1.6 and 2.0 times higher, respectively, compared to what was observed in the roots of the control plant (**Figures 5A, C**). Surprisingly, the L9 overexpressing line displayed similar levels of FM4-64 internalization than control plants although displayed higher levels of *SlSLSP6* transcripts. This result confirms the functional role of SlSLSP6 on endomembrane trafficking as a SNARE-like protein at the plasma membrane. It has been shown that salt stress induces endocytosis in root cells (Salinas-Cornejo et al., 2021). Therefore, we tested whether such a challenge would stimulate further membrane internalization in *SlSLSP6* overexpression lines. Then, tomato seedlings were subjected to 30 minutes of salt stress (200 mM NaCl) before the endocytosis rate was evaluated (**Figure 5B**). Indeed, salt treatment induced FM4-64 internalization on control plants (**Figure 5B**). Under this condition, the overexpressing lines L9 and L10 showed a higher internalization rate than control plants: 1.2 and 1.4, respectively, compared to 1.0 (**Figures 5B, D**). These results strongly suggest that the high level of *SlSLSP6* gene product resulted in modulation of vesicular trafficking increasing the rate of endocytosis playing an important role on the mechanisms by which plants manage the salt stress.

### 3.6 *SlSLSP6* overexpression induces sodium compartmentalization into the vacuoles

**Figure 6:**
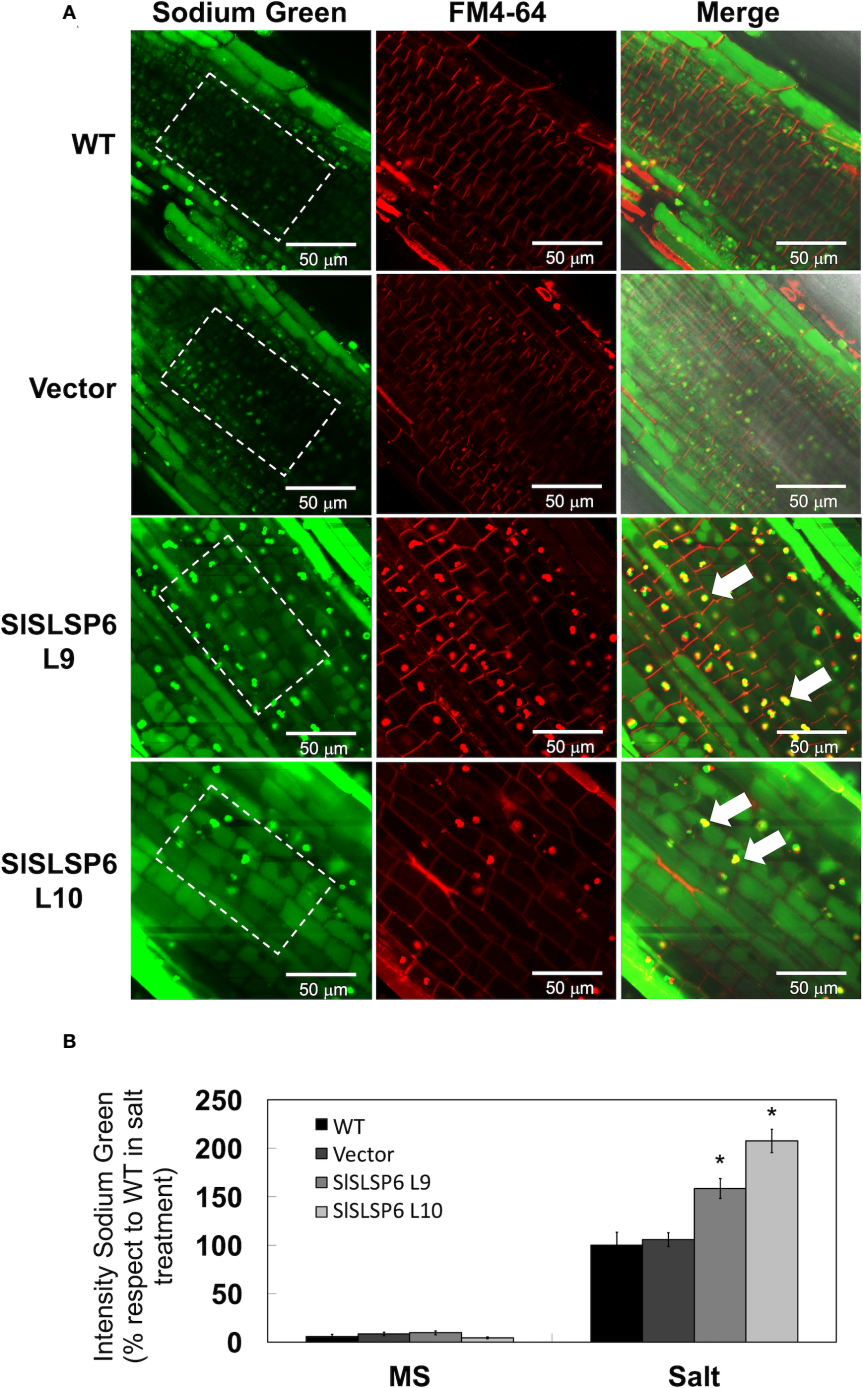
Sodium compartmentalization in *Solanum lycopersicum* root cell vacuoles under salt stress conditions. **(A)** Representative images of sodium accumulation in control tomato seedlings and lines overexpressing *SlSLSP6* under 200 mM NaCl and observed by confocal microscopy. The differentiation zone of the root was imaged. Tomato roots were treated with Sodium Green™ (green signal) and FM4-64 tracer (red signal). The bar indicates a scale of 50 µm. The ROI delimited by the white dashed lines was considered for quantification. **(B)** Quantification of fluorescence intensity of Sodium Green™ under normal conditions (MS) and salt stress (NaCl). The values ​​indicate the percentage relative to 100% fluorescence intensity evaluated in the WT plants under salt stress. Data indicate mean values ​​and standard errors. Asterisks indicate statistically significant differences between control lines and transgenic plants (p-value < 0.05), using one-way ANOVA.

An increase in the endocytosis rate promotes a greater compartmentalization of sodium in the vacuoles contributing to plant tolerance against salt stress conditions (Salinas-Cornejo et al., 2021). To verify this relationship, sodium accumulation was monitored using the Sodium Green™ fluorescent indicator in tomato roots of plants under normal conditions (**Supplementary Figure 6**) and subjected to 200 mM NaCl for 16 hours. Both *SlSLSP6* overexpressing lines L9 and L10 displayed a higher level of signal of Sodium Green than control plants (**Figures 6A, B**). Indeed, the L9 line displayed an increase of about 60% of fluorescent signal than control plants while L10 displayed the double amount of control plants. It was also observed that the accumulation of sodium occurs inside the vacuoles, both in the epidermal and cortical zone of the tomato roots, unlike what was identified in the control plants, where the accumulation is mainly in epidermal cells. Along with this, we observed that there was a strong accumulation of the sodium marker in structures with a pattern different from that of the vacuoles, whose composition and nature are unknown. These structures were also labeled with FM4-64 (white arrows **Figure 6A**). It could be speculated that they correspond to endosomal aggregates from the plasma membrane *via* the endocytic pathway, but this requires further investigation. Consequently, these results suggest that the greater compartmentalization of sodium in the vacuoles and in endosomal aggregates could be due to a greater endocytic dynamic of the transgenic plants that overexpress *SlSLSP6*, allowing to explain their enhanced tolerance under high salinity conditions.

## 4 Discussion

SNARE proteins are fundamental components in the vesicular trafficking machinery, playing a central role in the anchoring and fusion of vesicles with their acceptor membranes and in many biological processes, such as signaling in response to abiotic stress conditions (Leshem et al., 2006; Tarte et al., 2015; Nisa et al., 2017; Salinas-Cornejo et al., 2019; Won and Kim, 2020). They are relatively small proteins, in the range of 200-400 amino acids, where the SNARE domain is susceptible to form coiled-coil structures (Lipka et al., 2007). Most of the SNARE proteins described have transmembrane domains. However, some SNARE proteins are anchored to membranes through lipid modifications (Saito and Ueda, 2009). The homologous proteins of SNAP-25, a protein of the Qb+Qc-SNARE type, have a site in its sequence where a post-translational modification known as palmitoylation occurs, which would contribute to the stability of its association with the plasma membrane (Gonzalo and Linder, 1998; Greaves and Chamberlain, 2011; Won and Kim, 2020). In this sense, a *SNARE-like* gene, called *SlSLSP6* in tomato, has been identified and characterized, determining that the SlSLSP6 protein possesses a putative transmembrane domain, predicted as a palmitoylation signaling site. This type of reversible post-translational modification would allow a pivotal role in associations with membranes or interactions with other proteins, regulating their traffic or modulating their stability, anchoring proteins in the membrane in response to environmental conditions (Singh et al., 2016; Won and Kim, 2020). For another SNARE-like, it has been described the presence of sequences where lipid modifications occur, increasing the affinity of soluble proteins for membranes, allowing their insertion. Such is the case of SbSLSP from the halophyte plant *Salicornia brachiata*, in which a myristoylation site has been predicted (Singh et al., 2016). In addition, the predictive analysis of sites of putative lipid-type modifications on the SNARE-like sequence of *Arabidopsis thaliana* was performed, showing sites of possible palmitoylations or myristoylation (**Supplementary Figure 1**), which suggests that post-translational modifications increase the affinity of SNARE-like to the membrane or to certain proteins. In addition, the SlSLSP6 protein also presented a conserved domain associated with clathrins (**Supplementary Figure 1**), a protein complex that is involved in the assembly of clathrin coated vesicles (CCVs) budding from the plasma membrane. They are important in the selection and direction of cargo that will be transported. It has been described that these CCVs are part of the transport of membrane proteins through the endocytosis pathway (Reynolds et al., 2018). In this context, some plasma membrane proteins with typical SNARE-like features have been described in plants, including LOLITA (Longin-like protein interacts with TPLATE adaptor) from *A. thaliana* (Gadeyne et al., 2014) and SbSLSP (SNARE-like Superfamily Protein) from *S. brachiata* (Singh et al., 2016). LOLITA acts as an adapter protein together with other seven proteins, forming the TPLATE complex. This TPLATE complex directs clathrin-mediated endocytosis (Gadeyne et al., 2014; Yperman et al., 2021), is specific to plants and essential for their survival. However, its role under salt stress has not been described. On the other hand, SbSLSP has been associated with clathrin-mediated endocytosis regulation mechanisms and in response to salt and osmotic stress, as observed in transgenic tobacco plants that overexpressed *SbSLSP*, which improved their tolerance (Singh et al., 2016). Additionally, the analysis performed using the AGROBEST technique allowed us to observe the subcellular colocalization of the SlSLSP6 protein with different organelle markers (**Figure 4**). This analysis showed that this SNARE-like was located predominantly in the plasma membrane, supporting the *in silico* analysis. Also, we identified the presence of small structures in the vicinity of the plasma membrane, of unknown nature (**Figure 4**). The trans-Golgi network has been described as an essential hub from which multiple vesicle trafficking pathways branch, including the secretory and vacuolar pathway (Uemura et al., 2019). Although no colocalization was observed with VTI12, an EE/TGNE marker, it cannot be assumed that these unknown structures could correspond to endosomal aggregates that participate in a different pathway. Along with this, since the proteins SbSLSP and LOLITA are also located in the plasma membrane, the localization of SlSLSP6 in tomato could indicate the maintenance of the mechanism of action of this type of SNARE-like. Additionally, the relative expression analysis carried out for the identification of SNARE-like proteins in tomato (**Figure 1**) suggests that *SlSLSP6* could be playing an important role under salt stress conditions. Accordingly, genes such as *GsSNAP33* from *Glycine soja* (Nisa et al., 2017), *SlSNAP33* from *S. lycopersicum* (Salinas-Cornejo et al., 2021), *AtSFT12* from *A. thaliana* (Tarte et al., 2015) and *SbSLSP* from *S. brachiata* (Singh et al., 2016) are induced above basal levels of gene expression when plants are subjected to different salt concentrations and participate in the activation of tolerance mechanisms. Therefore, all these antecedents allow us to speculate that the function of this tomato SNARE-like, abundantly associated with the plasma membrane, could be generating an increase in vesicular traffic and modulating clathrin-mediated endocytosis, being a candidate capable of contributing to the response of plants to adverse conditions, such as salinity stress.

This investigation revealed that *SlSLSP6* overexpression improved the physiological and biochemical parameters of tomato plants subjected to salt stress. In previous studies, it was observed that wild-type tomato plants subjected to 300 mM NaCl presented evident symptoms of stress around 15 days, decreasing the relative water content and photosynthesis, which was attributed to a disruption of normal functioning and maintenance of the cell structure (Orellana et al., 2010). The salt treatment caused a constant and marked inhibition of the photosynthetic machinery in the control plants, unlike what was observed in the *SlSLSP6* overexpressing lines, which maintained proper functioning and demonstrated a better photosynthetic rate (**Figure 2G**). The overexpression of *G. soja GsSNAP33* in *A. thaliana* plants also generated tolerance under abiotic stress conditions, where the transgenic genotypes presented a higher survival rate, higher biomass production and a higher photosynthetic rate (Nisa et al., 2017). Under salt stress or due to water deficiency, a closure of the stomata occurs, which limits the concentration of CO_2_ and damages the photochemical machinery of the leaves, strongly depressing photosynthesis (Chaves et al., 2009; Keisham et al., 2018). In addition, it was observed that *A. thaliana* plants that overexpressed *AtSFT12* presented a higher fresh weight and greener leaves compared to control plants, when they were subjected to salt stress conditions (Tarte et al., 2015). This suggests a change in the distribution of some proteins that reside in the plasma membrane, such as aquaporins, which could be involved in the reduction of intracellular water loss (Baral et al., 2015b). Likewise, a hyperosmotic condition disorganizes the balance of cell membranes. Cell volume decreases, which leads to an excess of plasma membrane that must be endocytosed to reestablish cell turgor (Zwiewka et al., 2015; Zhang et al., 2019). Vesicular traffic is the indispensable process in the life of plants, which allows the movement of cellular materials inside small vesicles. Specifically, endocytosis plays a fundamental role in the removal and/or relocation of proteins, lipids, and other materials from the plasma membrane (Baral et al., 2015b; Dragwidge and VAN Damme, 2020). It has been reported that during salt stress there is an increase in endocytosis in *A. thaliana* (Baral et al., 2015a). In this investigation, an increase in endocytosis was evidenced in the overexpressing tomato lines, compared to the control plants, considering both normal and salt stress conditions (**Figure 5**). Other evidence indicates the consistency of these results, as is the case of salt stress tolerant plants that overexpress *SchRabGDI1* (San Martín-Davison et al., 2017) or *SlSNAP33* (Salinas-Cornejo et al., 2021), suggesting an acceleration of endocytosis in the roots of tomato plants overexpressing *SlSLSP6*. Similarly, it is hypothesized that overexpression of *SbSLSP* from *S. brachiata* would increase clathrin-mediated endocytosis, improving the stability and integrity of membranes and modifying the distribution of transporters in the plasma membrane (Singh et al., 2016). The increase in endocytosis observed in the transgenic lines could be contributing to the internalization of the plasma membrane, to restore the turgidity of the cells (Zwiewka et al., 2015), and to the removal of proteins from the plasma membrane, such as aquaporins, to allow osmotic adjustment within cells. This mechanism would be activated under salt stress conditions and other types of abiotic stress, improving tolerance in tomato plants.

To mitigate the consequences produced by salt stress at the physiological, cellular and molecular level, plants respond by activating different mechanisms. A high concentration of Na^+^ ions will cause an ionic imbalance, triggering a massive entry of toxic ions into the cells and will also produce changes at the lipid level (Baral et al., 2015b; Orlova et al., 2019). Along with this, salinity stimulates the accumulation of ROS, which in high concentrations are toxic to cells. At low concentrations, these molecules help mitigate salt stress through a series of signal transduction mechanisms (Mangal et al., 2022). The evidence obtained in this investigation indicated that the overexpressing lines showed less damage at the level of cell membranes (**Figure 2H**). Previous studies have reported that tobacco plants that overexpress *SbSLSP* present lower levels of MDA in response to stress (Singh et al., 2016). Related to this, the overexpression of *SlSNAP33* has also been shown to provide tolerance to salt stress in tomato plants presenting less damage to cell membranes (Salinas-Cornejo et al., 2021). When plants are subjected to hyperosmotic conditions, caused by salt stress for example, the response of the cells is associated with the production of ROS and the compartmentalization of toxic ions in the vacuoles, between other mechanisms (Zwiewka et al., 2015). Here, using the H_2_DCFDA fluorescent tracer, it has been shown that the overexpression of *SlSLSP6* in tomato plants causes a significant reduction in hydrogen peroxide, observed mainly in the L10 transgenic line (**Figure 3**). This response has also been reported in tomato plants that overexpress *SlSNAP33*, which has been associated with the endocytosis of plasma membrane NADPH oxidases, generating a lower production of ROS in root cells (Salinas-Cornejo et al., 2021). In addition, in *A. thaliana* it has also been reported that the internalization of NADPH oxidases, such as RbohD, causes the production of ROS inside vesicles, decreasing its levels in cells (Leshem et al., 2006). This is interesting, since this decrease in the accumulation of ROS in the vacuoles would generate a greater compartmentalization of vacuolar sodium when plants are subjected to salt stress. It is suggested that there would be a decrease in oxidative damage in the tonoplast and in v-ATPases, helping to maintain the proton gradient and promoting the sequestration of cytoplasmic sodium inside the vacuole (Leshem and Levine, 2007). In this context, the results obtained showed that the transgenic plants had a greater accumulation of sodium in the vacuoles compared to the control plants (**Figure 6**). Similar results were presented by Tarte et al., 2015 and Salinas-Cornejo et al., 2021, where it was shown that the *AtSFT12* overexpressing plants of *A. thaliana* subjected to 100 mM NaCl and the *SlSNAP33* overexpressing tomato plants subjected to 200 mM NaCl compartmentalized more sodium in the vacuoles, which was monitored with the fluorescent tracer Sodium Green™. Interestingly, along with sodium accumulation in the vacuoles, Sodium Green™ staining was observed in rounded compartments within the cells of unknown nature (**Figure 6**). Since these structures were stained with the membrane marker FM4-64, it could be assumed that they are bordered by a lipid membrane. These compartments were observed in plants subjected to salt stress, stained with both FM4-64 and Sodium Green™. In overexpressing plants, it seems that these structures are accumulating more sodium than in WT plants. It could be speculated that these compartments have characteristics similar to endosomal or prevacuolar bodies, which would be sequestering sodium ions from the cytosol, mediated by the Na^+^/H^+^ antiporters NHX5 and NHX6, which reside in mobile endosomal structures (Bassil et al., 2011). Similar characteristics were described by Hamaji et al., 2009, where they observed the formation of vesicles that surrounded the vacuoles in *A. thaliana* root cells. These vesicles were marked with the fluorescent tracer Sodium Green™, which allows us to assume that it is sodium accumulation inside these compartments. In any case, to give certainty to these speculations, more research is required. Therefore, from this research arise the approaches that imply that an acceleration of endocytosis would occur, activating the removal of transporters from the plasma membrane. Because of the above, an increase in exocytosis would be generated, allowing the replacement of damaged lipids from the membrane. Moreover, the traffic towards the vacuole would increase, removing the excess sodium from the cytosol in a greater proportion to avoid its intracellular toxic effects, to finally obtain an improved tolerance of *SlSLSP6* overexpressing tomato plants to salt stress.

## 5 Conclusion

Here, we identified and characterized a *SNARE-like* gene, called *SlSLSP6* in tomato, determining its possible relationship with tolerance to salt stress. We can speculate that the function of this tomato SNARE-like fulfills its role in the plasma membrane, could be increasing the vesicular traffic and modulating clathrin-mediated endocytosis. This increase in endocytosis could be impacting in the replacement of lipids and in the removal of proteins from the plasma membrane. Furthermore, the traffic towards the vacuole would increase, removing the excess of toxic ions from the cytosol. These mechanisms would be activated against salt stress, improving the physiological and biochemical parameters of tomato plants. Finally, these results provide the knowledge to understand the role of this type of SNARE-like protein during salt stress and could be a suitable candidate to contribute to breeding programs to improve tolerance in tomato plants.

## Data availability statement

The original contributions presented in the study are included in the article/**Supplementary Material**. Further inquiries can be directed to the corresponding author.

## Author contributions

Conceived and designed experiments, JS-C, JM-E, IV, and SR-L; writing—original draft preparation, JS-C, JM-E, LN, and SR-L; writing—review and editing, JS-C, JM-E, LN, and SR-L; visualization, JS-C; project administration and funding acquisition, SR-L. All authors contributed to the article and approved the submitted version.
