# Supplementary material for: A SNARE-like protein from Solanum lycopersicum increases salt tolerance by modulating vesicular trafficking in tomato
Source: Front Plant Sci. 2023 Aug 1;14:1212806. doi: 10.3389/fpls.2023.1212806 (PMC10431929; doi:10.3389/fpls.2023.1212806)
Supplement: Supplementary file 1 [file DataSheet_1.pdf]

Supplementary Material

1 Supplementary Figures and Tables

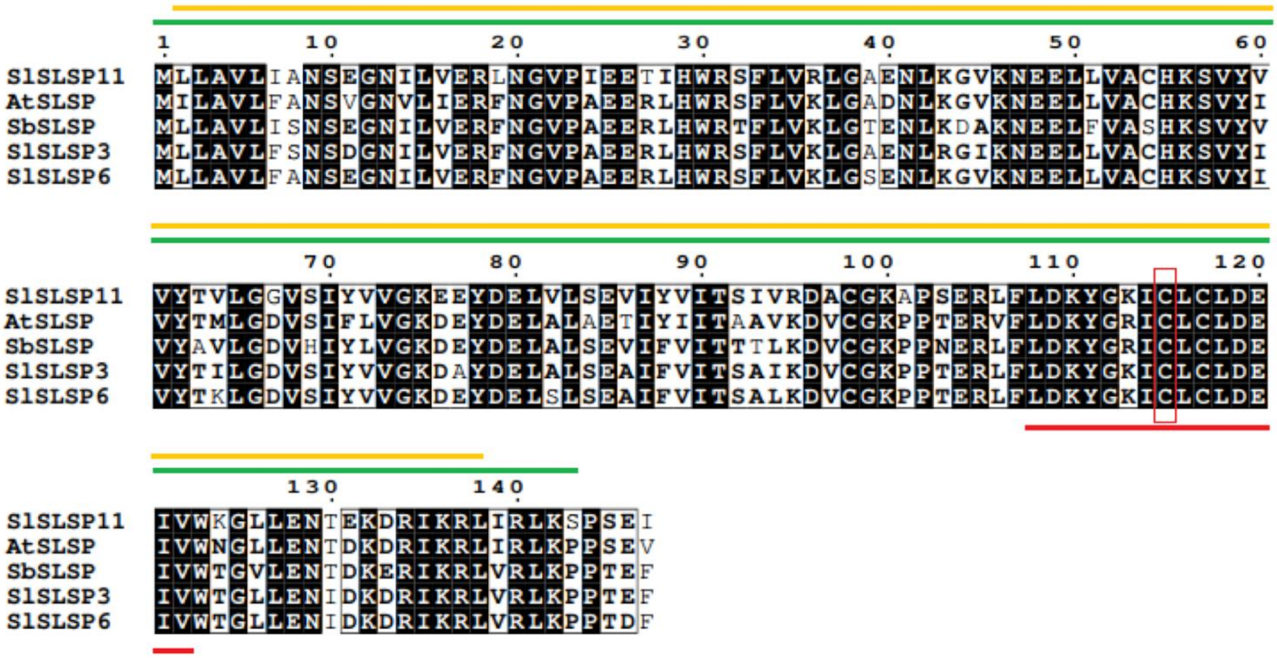

Supplementary Figure 1. Multiple sequence alignment of SlSLSP6 and SNARE-like proteins. SNARE-like sequences from *Arabidopsis thaliana* (AtSLSP), *Salicornia brachiata* (SbSLSP) and *Solanum lycopersicum* (SlSLSP6, SlSLSP3 and SlSLSP11) were aligned using MUSCLE software. Black boxes show identical residues. The clathrin vesicle coat proteins interactor domain "Clat_adaptor_s" is shown in orange from the position 2 – 138 of SlSLSP6. The SNARE-like superfamily domain "Longin-like" is present from amino acid 1 to the 143 (green line). The Cys115 is predicted as a putative palmitoylation residue. The red line denotes a domain that may interact with membranes.

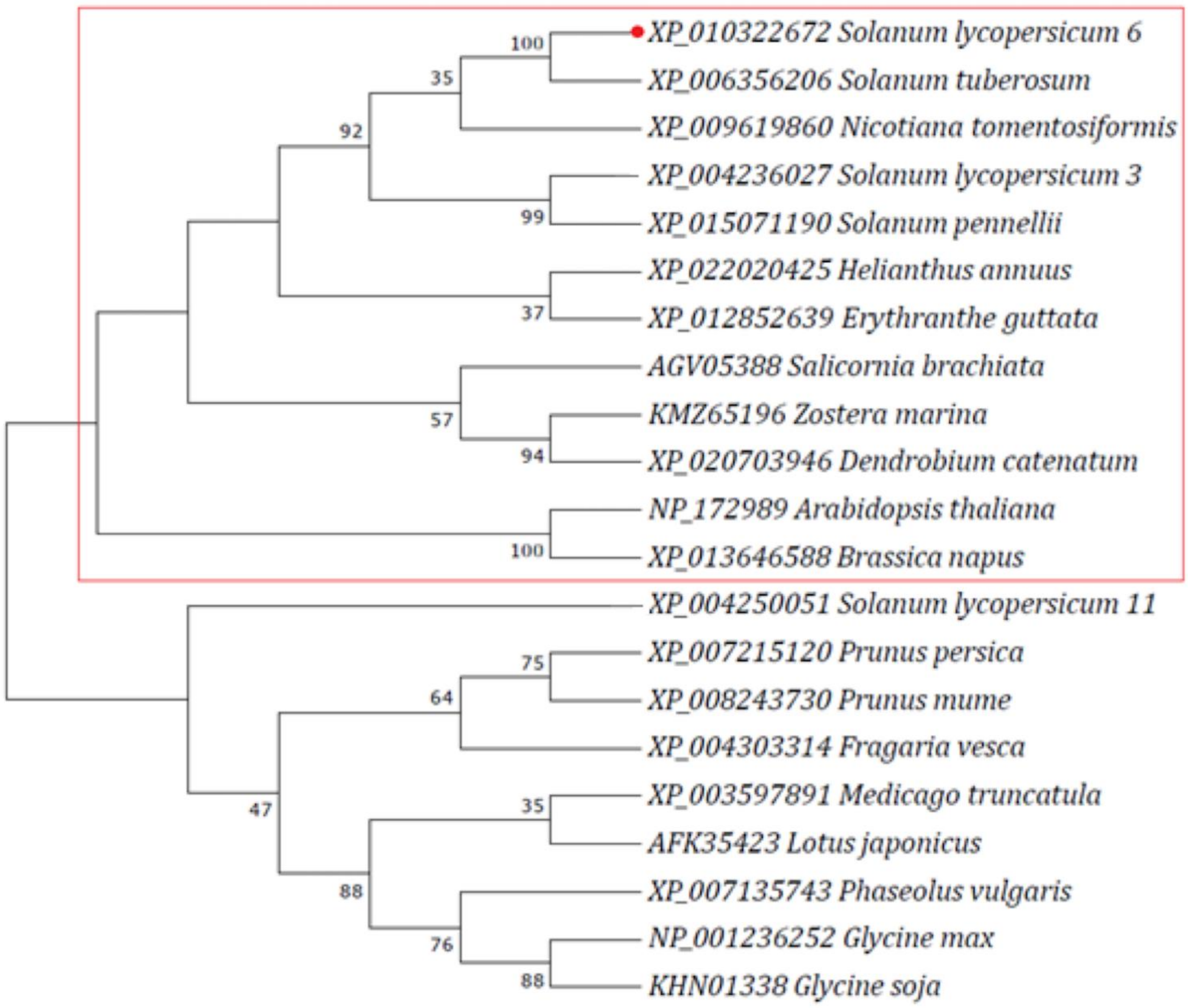

Supplementary Figure 2. Phylogenetic tree of SNARE-like protein from plant species. Solgenomics and NCBI databases were used as protein sequence search engines. The phylogenetic tree was constructed using the MEGA7 with the neighbor-joining method and bootstrap analysis of 1000 replications. SlSLSP6 cluster with SNARE-like proteins from tomato species and salt tolerant plants. The annotation of each protein included in the phylogenetic tree corresponds to the accession number of the sequences in NCBI, followed by the species to which each sequence belongs.

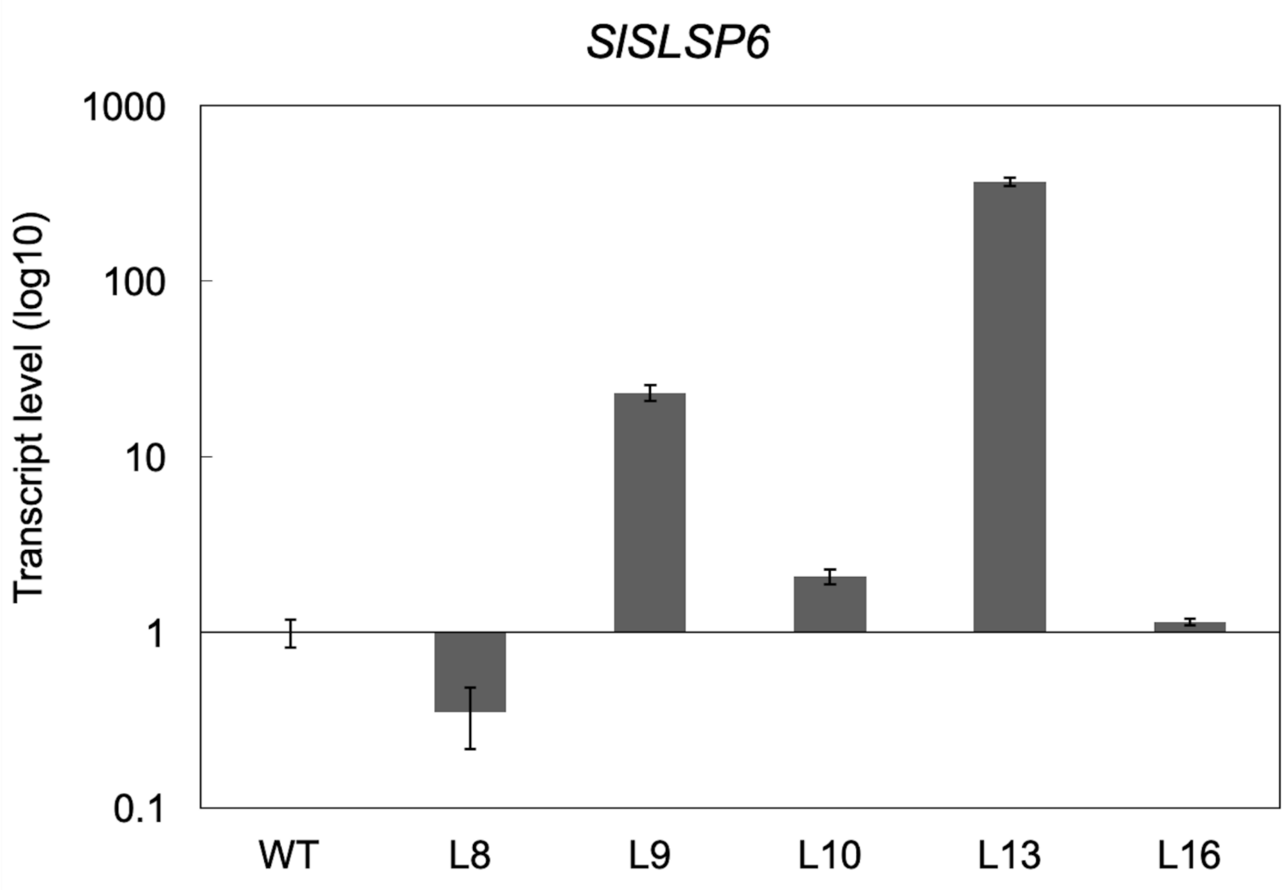

Supplementary Figure 3. Transcript levels of *SlSLSP6* in overexpressor tomato plants (*S. lycopersicum*). Transcript levels of *SlSLSP6* were determined by qRT-PCR analysis in transformant tomato plants. Results are expressed and log10. Mean and standard deviation of three biological replicates and three technical replicates are shown.

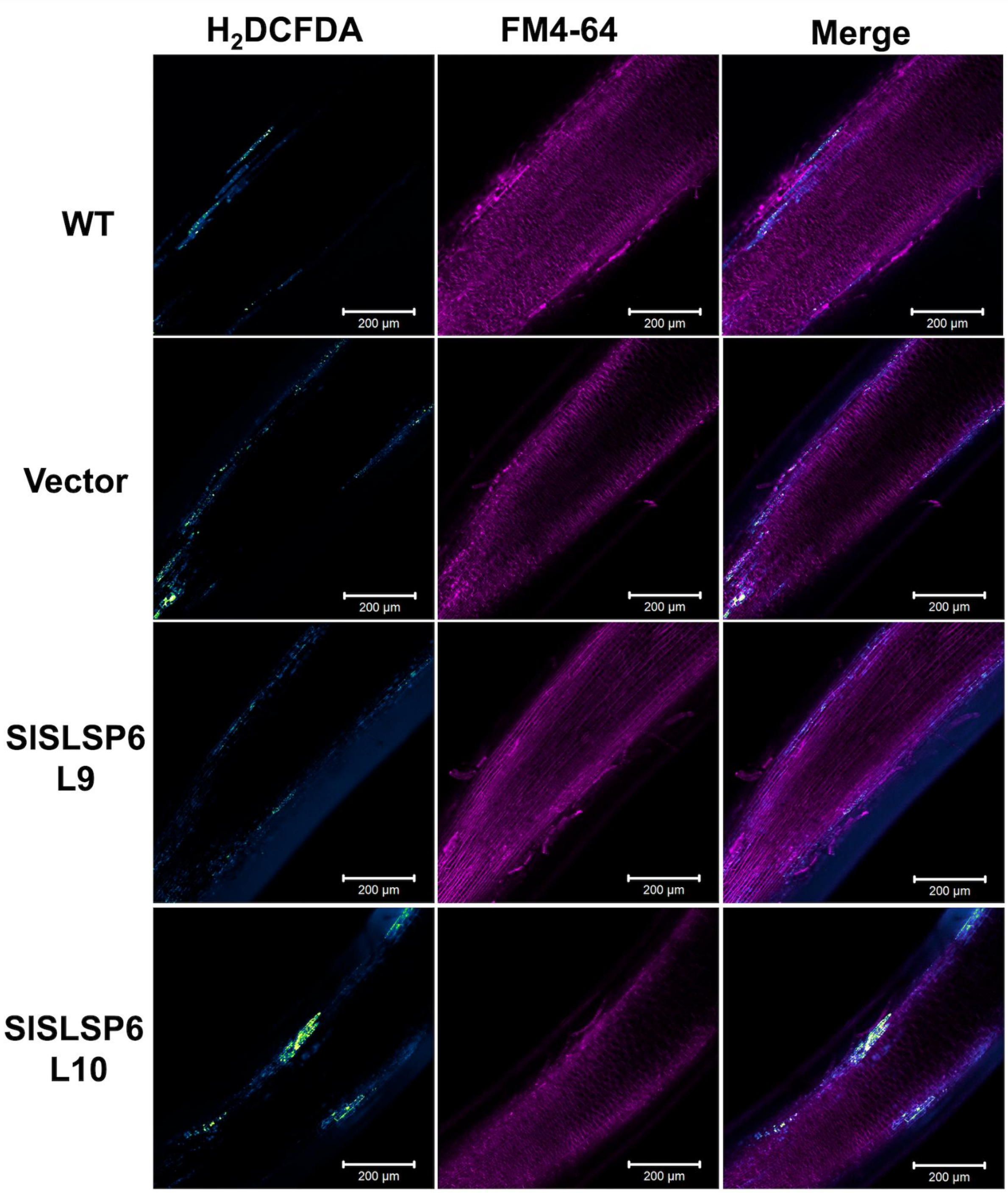

Supplementary Figure 4. H_2O_2 content in *Solanum lycopersicum* roots under normal conditions. Representative images of H_2O_2 production in control tomato seedlings and *SlSLSP6* overexpressing lines, observed by confocal microscopy. Tomato roots were treated with H_2DCFDA (color-coded intensity, with the lowest fluorescence intensity corresponding to blue and highest fluorescence intensity corresponding to green color) and FM4-64 tracer (in magenta). The bar indicates a scale of 200 µm.

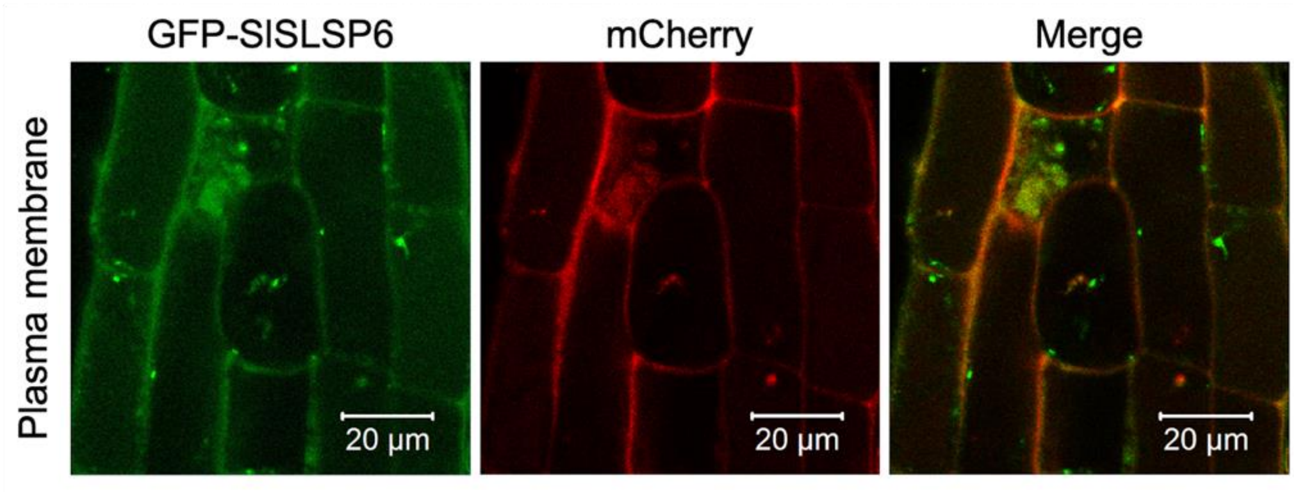

Supplementary Figure 5. The SlSLSP6 protein is localized to the plasma membrane. 10-day-old Arabidopsis thaliana PIP1;4-mCherry lines were transiently transformed with the vector that allows to express a GFP-fusion to the SlSLSP6 version. GFP and mCherry fluorescence in root cells were visualized by confocal microscopy.

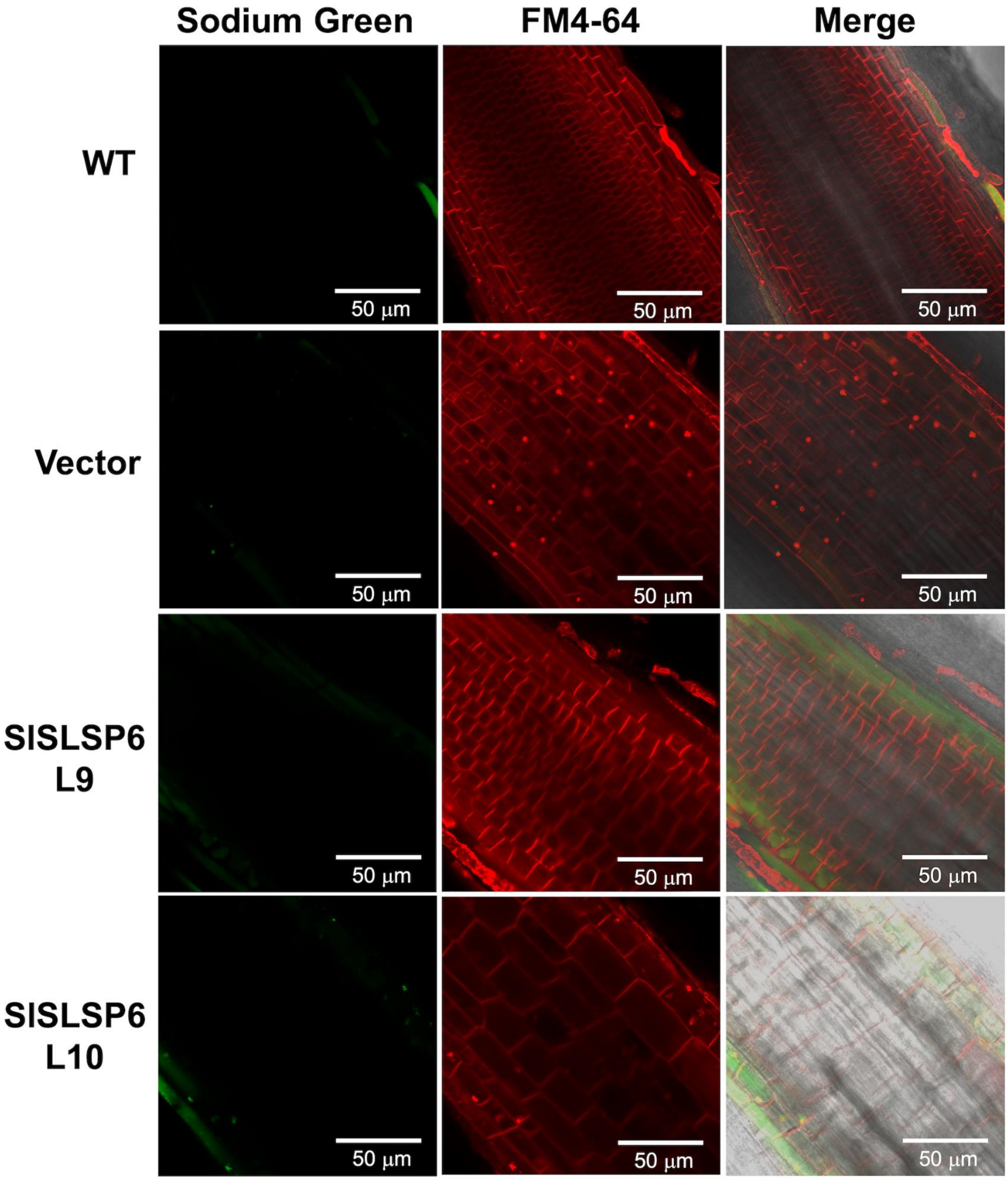

Supplementary Figure 6. Sodium accumulation in *Solanum lycopersicum* roots under normal conditions. Representative images of sodium detection in control tomato seedlings and *SlSLSP6* overexpressing lines, observed by confocal microscopy. Tomato roots were treated with Sodium Green (green signal) and FM4-64 tracer (red signal). The bar indicates a scale of 50 µm.

Supplementary Table 1. List of primers used in this work.

Gene	Sequence (5' – 3')	A. Temp (°C)
**Primers for qRT-PCR**		
**SLSP6**	Fw: 5'-AAAAATGCTGCTAGCGGTGC-3'<br>Rv: 5'-AAAAGACCGCCAATGCAGAC-3'	58
**Ubiquitin3**	Fw: 5'-GAAGAAGAAGACCTACACCAAGCC-3'<br>Rv: 5'-CACTCCTTACGAAGCCTCTGAAC-3'	58
**AREB1**	Fw: 5'-CAGGTTTAATGGCTGGTAGTATCCC-3'<br>Rv: 5'-GCTGTGATTGTTGGTTCTGTTGC-3'	58
**TSW12**	Fw: 5'-CTCCACGGTTCAGTAAAG-3'<br>Rv: 5'-CCATAAGCAGGATCACAC-3'	58
**Primers for ORF amplification**		
**SlSLSP6**	Fw: 5'-TTCTAGAATGCTGCTAGCGGTGCTATT-3'<br>Rv: 5'-TGAGCTCTCAGAAATCGGTGGGTG-3'	58
**Primers for subcellular location**		
**GFP-SlSLSP6**	Fw: 5'-TCTAGAAATGCTGCTAGCGGTGC-3'<br>Rv: 5'-GGATCCTCAGAAATCGGTGGGT-3'	58
